# Physical Modeling of the Impeller Construction Impact on the Aluminum Refining Process

**DOI:** 10.3390/ma15020575

**Published:** 2022-01-13

**Authors:** Mariola Saternus, Tomasz Merder

**Affiliations:** Faculty of Materials Engineering, Silesian University of Technology, Krasinskiego 8, 40-019 Katowice, Poland; tomasz.merder@polsl.pl

**Keywords:** aluminum, refining, rotary impeller, physical modelling

## Abstract

Obtaining high-quality aluminum is associated with the use of an effective method of refining, which is argon-purging, in which gas bubbles are introduced into the liquid metal by means of rotary impellers. Various rotary impellers are used in the industry; however, if a newly designed impeller is constructed, it should be tested prior to industrial use. For this purpose, physical modeling is used, which enables the investigation of the phenomena occurring during refining and the selection of optimal processing parameters without costly research carried out in the industry. The newly designed rotary impeller was tested on the physical model of a URO-200 batch reactor. The flow rate of refining gas was: 10, 15 and 20 dm^3^·min^−1^, whereas rotary impeller speed was 300, 400 and 500 rpm. The research consists of a visualization test showing the schemes of the gas bubbles’ dispersion level in the liquid metal and experiments for removing oxygen from water, which is an analogue of removing hydrogen from aluminum.

## 1. Introduction

Currently, there is a steady increase in aluminum production due to the growing demand for aluminum products. Obtaining a high purity of aluminum and its alloys became a basic technological steps. It is used both in the production of primary aluminum (from bauxite) and secondary aluminum (from scrap). Contaminating elements such as hydrogen, alkali metals, alkaline earth metals and inclusions like oxides, borides, carbides and chlorides can be introduced into the liquid metal from a raw alumina feed, electrolyte cell, anode, refractory cells and furnaces and the atmosphere causing serious defects if they exceed the assumed safe level. Therefore, the main goal of refining is to remove unwanted hydrogen from liquid metal, which is the main reason for the porosity, leading to a deterioration of the strength properties of finished aluminum products [1,2,3,4,5].

The methodology used to remove impurities from aluminum is based on mechanical mixing devices, as well as on devices for gas injection. Gas bubbles can be generated by various types of nozzles, porous plugs fixed in the bottom of the reactor, lances or rotating impellers [6,7,8,9]. Among others, thanks to the promising results of mixing and injection processes, the most popular method of introducing an inert gas into the molten metal is a rotor ending with an impeller (e.g., ACD, AFD, Alpur, ASV, GBF, GIFS, LARS, RDU, URO-200). Impellers are generally classified into two types: pump type and propeller type. In the pump impeller, the gas is introduced into the metal in the open head chamber, and then the resulting mixture is pumped through the outflow holes. Such impeller allows the introduction of larger amounts of gas, but requires a higher impeller rotary speed. In a propeller-type impeller, the refining gas flows through a system of channels and is ejected through small holes on the circumference of the impeller. The impeller design has a large impact on the shape and size of the generated gas bubbles. In addition, a properly designed impeller ensures a long service life—even up to 1000 refining cycles. Rotating impeller constructions used in industrial practice are most often the subject of patent claims, which means that copying them is illegal. New design solutions for these refining reactor components, whose main objective is to improve the refining performance and reduce process costs, must therefore be original. This requires advanced scientific research in both industrial and laboratory conditions. One of the research methods used in this field is physical modeling [10,11,12,13,14,15].

Physical modeling as well as numerical modeling have become popular tools for observing and better understanding processes occurring during smelting and refining processes commonly used in the steel and non-ferrous metallurgy [16,17,18,19,20,21,22]. The research focuses mainly on the nature of the flow and the circulation of bubbles that are directly related to the working conditions.

Research with the use of physical models requires the compliance with the rules resulting from the principles of similarity, which refer to the characteristic features of a real object and which have a significant impact on the phenomena occurring in the examined process. The conditions of similarity are of a geometric, mechanical, kinematic, thermal or chemical nature. In order to ensure the similarity of the studied phenomena, one should strive to achieve a full similarity, which in real conditions is often difficult to implement, and therefore the dominant quantity in the process under study is usually selected. In the case of model tests of the argon injection process into aluminum, it is necessary to meet the principles of geometric similarity between the model and the real object and the hydrodynamic similarity for the flow of liquid in the model and object, i.e., kinetic, dynamic and thermal similarity [23,24,25,26]. In order to keep the mentioned rules of similarity, it is enough to fulfill them if the appropriate number of criteria for the model and the tested object are equal, and the results obtained as a result of experiments carried out on physical models can be transferred to real conditions. In the case of the physical modeling of the aluminum refining process, the most important criterion numbers are the Reynolds, Weber and Froude numbers. The Reynolds number characterizes the hydrodynamic similarity of the fluid flow, which is the criterion of turbulence of the flow. The Froude number characterizes the effect of gravity on fluid flow phenomena and it is the dominant criterion in the aluminum refining process, which means that the values of the Froude number should be equal in real conditions and in the physical model. Very often, in the case of aluminum refining, the Weber number is also calculated, i.e., the ratio of the inertial forces to the surface tension. Water is usually used as the medium in physical modeling. The factors affecting the selection of water are: its availability, its relatively low costs and ecological reasons; but the most important thing is that some properties of water, especially kinematic viscosity at room temperature, are very close to those of liquid aluminum [26]. The difference in the density of water and liquid aluminum will not play a role here, because in the formula for the Froud criterion number, which is the dominant criterion in the construction of models in the refining process, there is kinematic viscosity.

In the case of the degassing of aluminum, the research on physical modeling focused, in particular, on determinant flow patterns, such as the flow in geysers or columns, minimal dispersion, intimate dispersion and uniform dispersion [9,27,28,29]. Camacho-Martinez et al. [30] and Mi et al. [14] investigated the operation of various impellers’ design and their position, while Tovio et al. [11] measured the size of bubbles and the distribution of gas bubbles. Camacho-Martinez et al. [30], Hernandez-Hernandez et al. [14] and Saternus and Botor [31] conducted studies on the removal of oxygen from water as an analogue of hydrogen removal from aluminum. Mainly, all researchers tested different processing parameters, such as the refining gas flow rate and impeller speed. Therefore, based on a review of the literature [12,15,29,32] and own experiences with different types of impellers [10,13,28,33], a new impeller was designed [34]. In the presented research, the degree of gas bubble dispersion in the modeling liquid and the removal of oxygen from water were analyzed at various technological parameters (gas flow, impeller speed). The obtained results for the designed impeller were compared with three others.

## 2. Research Study and the Object

**Figure 1:**
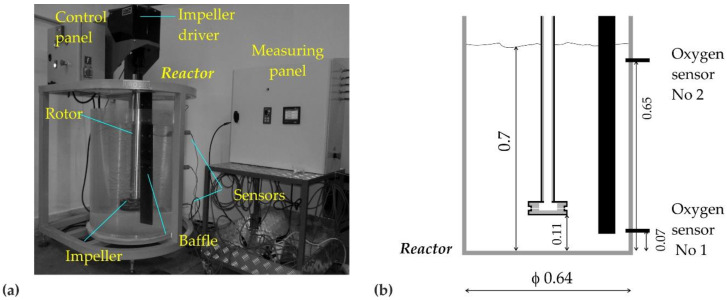
(**a**) View of the URO-200 model; (**b**) scheme model with marked places of fixed measuring sensors and the most important dimensions (in m).

Physical modeling tests were carried out on the URO-200 reactor model (see Figure 1), made at IMN-OML in Skawina and mapping industrial equipment. This model is equipped with a precise device controlling the amount of gas introduced and regulating the rotation. It is based on the similarities between a geometric model and an industrial object, as well as on the hydrodynamic similarity to liquid flow in a model and an industrial device based on the compliance with the criterion number of Froude, as was stated above [23,24]. Liquid aluminum is simulated by water due to the fact that the kinematic viscosity of water and liquid aluminum is very close. Table 1 presents a comparison of the basic parameters of liquid aluminum and water and the calculated values of criterion numbers (Reynolds, Froude and Weber) for water (in 293 K) and aluminum (in 973 K).

**Figure 2:**
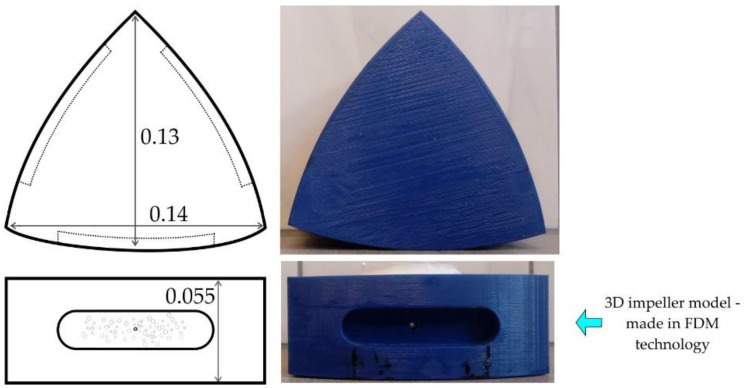
Schematic diagram of the analyzed impeller with its main dimensions, m.

Figure 2 shows a schematic diagram of a designed impeller that is undergoing modeling testing. It is characterized by straight edges, which should promote a good mixing of gas bubbles in the modeling liquid, and consequently the desired uniform dispersion of gas bubbles in the whole volume of the refining reactor. The designed impeller has a different shape than the commonly used one and is characterized by a simple (compact) structure, which should have a positive effect on the length of its operation (wear). The impeller’s design was create in the SolidWorks program, and the model was printed using FDM (Fused Deposition Modeling) 3D printing. A PLA (Polylactic Acid) filament was used as the working material. The design and preparation of the impeller have been described in detail in [30].

The aim of the study was to analyze the operation of the impeller, taking into account a better efficiency of the hydrogen removal from liquid aluminum during the refining process. Qualitative and quantitative experimental tests were carried out on a physical model. The qualitative research was based on the visualization of the gas bubble dispersion in the modeling liquid, while the quantitative tests consisted in measuring the dissolved oxygen content in water and the changes in oxygen concentration during argon purging. Before each experiment, the water was saturated with gaseous oxygen to a value of 25 mg·dm^−3^. The experiment began when an inert gas (argon) was introduced by the impeller during its rotation. At the same time, a measuring session was started, which means recording the dispersion image with digital cameras and recording the changes in the dissolved oxygen content in the modeling liquid (water) at the relevant points of the model (see Figure 1b). It was assumed that the experiment would be run for 10 min (600 s) in order to compare the obtained values. This time is equivalent to the time of refining carried out in industrial conditions on the URO-200 device. The obtained data were archived in the computer’s memory and subjected to further digital processing. The research methodology developed in this way allowed us to conduct a series of experiments in the unchanged conditions of the model’s functioning.

Three to five experiments were carried out for each measurement session. Table 2 presents variants of the experiments carried out for processing parameters (gas flow rate and impeller speed), which were selected based on those commonly used in industrial practice. As a consequence, such a selection of processing parameters gives us the opportunity to compare the results for the tested impeller with others used in the industry.

**Figure 3:**
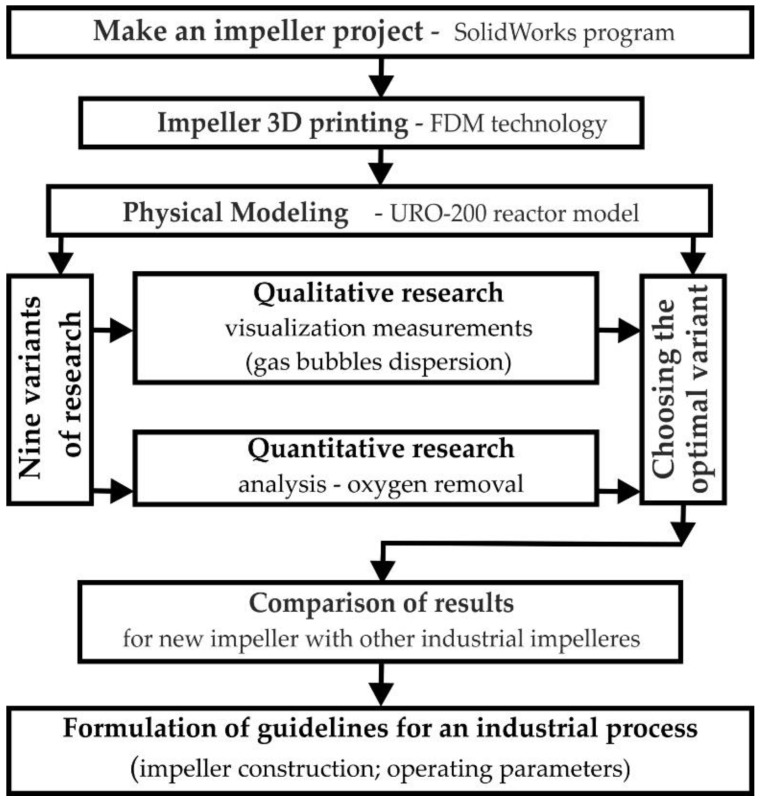
Research plan diagram.

Due to the wide scope of the tests and the last stage of comparing the quality of the impeller structure with other industrial applications, a test plan (Figure 3) was prepared before starting the tests, which presented the work’s next stages.

## 3. Results and Discussion

**Figure 4:**
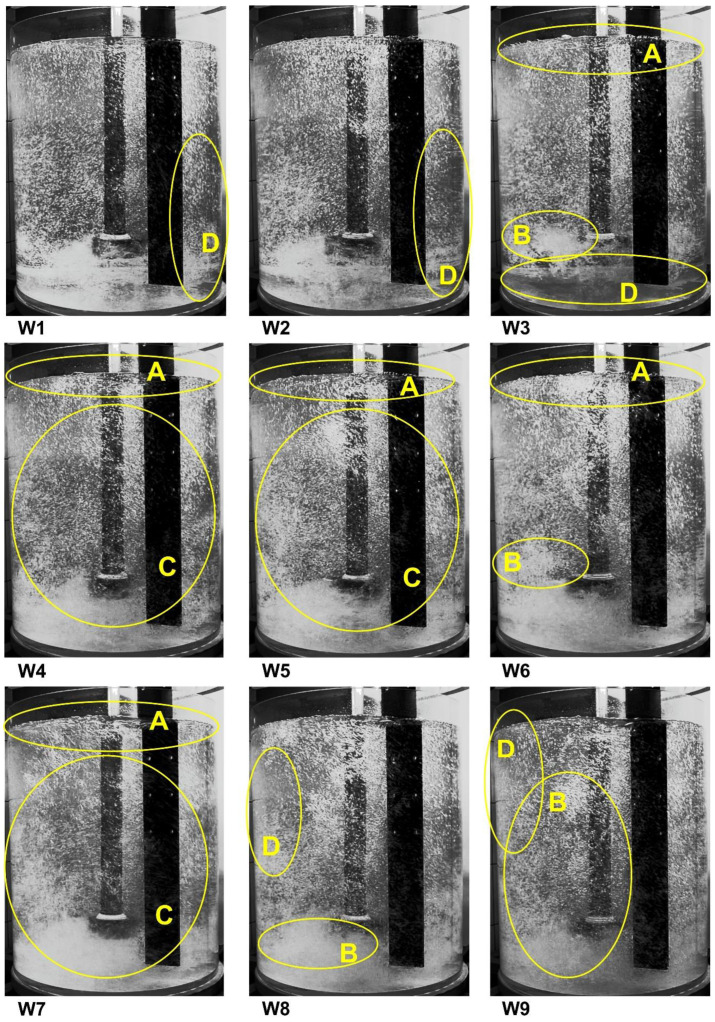
Gas bubbles’ dispersion registered for different processing parameters, A—single gas bubbles formed on the surface of the modeling liquid, B—excessive formation of gas chains and swirls, C—uniform distribution of gas bubbles in the entire volume of the tank, D—dead zones without gas bubbles, no dispersion.

**Figure 5:**
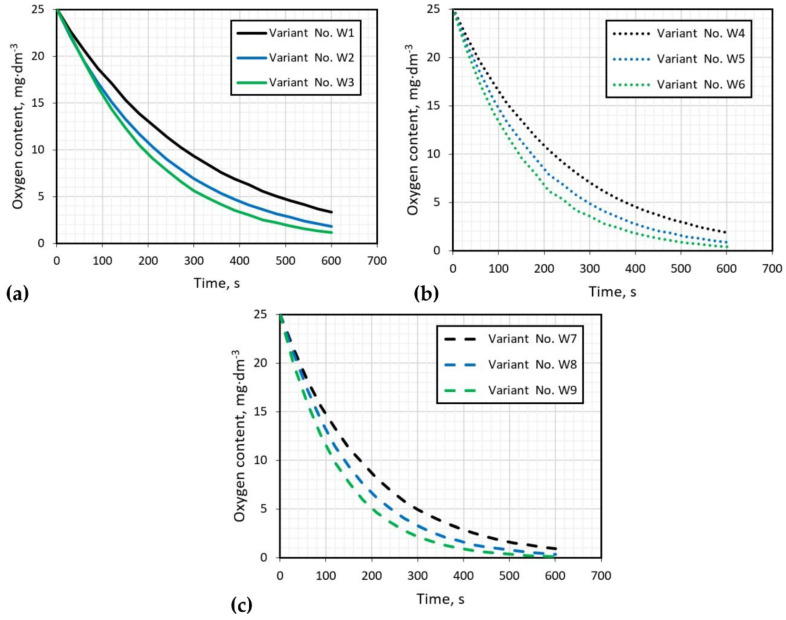
Results of oxygen removal, impeller speed: (**a**) 300 rpm, (**b**) 400 rpm, (**c**) 500 rpm.

Figure 4 presents the results of the visualization measurements for the tested processing parameters. Before being used in the industry, each impeller must be tested in laboratory conditions, in water models. The most important process parameters for aluminum refining in cyclic conditions are the gas flow rate and the rotary impeller speed. These two parameters allow us to achieve the desired uniform dispersion with the optimal impeller geometry, i.e., fine gas bubbles are evenly distributed throughout the liquid volume. By analyzing the variants presented in Figure 4, the best processing parameters of the rotor operation can be determined. As already mentioned, there are four patterns of gas bubble dispersion in the modeling fluid. The first one, called geyser flow or column flow, is characterized by the generation of large gas bubbles rising only near the impeller shaft. This case yields the worst degassing results. In the variants presented in Figure 5, no such dispersion was observed. The next minimum dispersion is characterized by a better distribution of gas bubbles in the volume of water. However, there is still no dispersion in the bottom and side walls. This case was also not observed.

The next two cases are the intimate dispersion and the uniform dispersion. The most desirable is an uniform dispersion due to the dispersion of gas bubbles in the entire volume of the modeling liquid. In the case of an intimate dispersion, there is no dispersion in some parts of the volume of the reactor, for example in the lower part. There is also a fifth pattern, called excessive dispersion or flow chain formation, that is characterized by the formation of vortices and a chain of bubbles that can cause hydrogen to be reintroduced into the liquid metal. To better distinguish the type of dispersion on Figure 4, some parts of the reactor volume were marked according to the following information:

- A—single gas bubbles formed on the surface of the modeling liquid,
- B—excessive formation of gas chains and swirls,
- C—uniform distribution of gas bubbles in the entire volume of the tank,
- D—dead zones without gas bubbles, no dispersion.

Based on the results from Figure 4, the visualization results showing the dispersion types for all tested variants could be summarized in Table 3.

Figure 5 presents the results of the oxygen removal from water carried out for all tested variants. The experiment was carried out until the oxygen concentration was below 1 mg·dm^−3^. The graphs in Figure 5 show the results during a 10-min (600 s) refining interval to compare the achieved process parameter values. This time is equivalent to the time of refining carried out in industrial conditions on the URO-200 device. The worst results of oxygen removal, at a level of 2 to 3 mg·dm^−3^, are obtained for variants W1 (300 rpm, 10 dm^3^·min^−1^), W2 (300 rpm, 15 dm^3^·min^−1^) and W4 (400 rpm, 10 dm^3^·min^−1^). Other variants obtained results lower than 1 mg·dm^−3^ of oxygen content after argon purging. However, if there is excessive dispersion in variants W8 and W9, the best variants appear to be W5 (400 rpm, 15 dm^3^·min^−1^) and W7 (500 rpm, 10 dm^3^·min^−1^).

Table 4 presents the refining time that corresponds to the total time required to eliminate 90% of the dissolved oxygen according to the expression cited in [12]. The refining time for the technological parameters considered was from 270 to 690 s. The smallest time was observed for variant W9 (500 rpm, 20 dm^3^·min^−1^), but in this case the excessive dispersion was observed. For variants W5 (400 rpm, 15 dm^3^·min^−1^) and W7 (500 rpm, 10 dm^3^·min^−1^), characterized by a uniform dispersion, the refining time was the same in both cases—420 s.

## 4. Comparison of Results for a New Impeller with Other Industrial Applications

**Figure 6:**
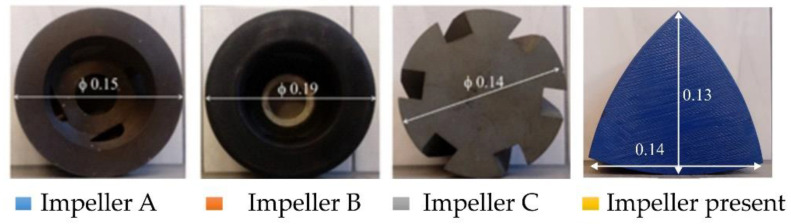
View of the four impellers for which the result of the refining time needed to remove 90% of dissolved oxygen was compared, dimensions in m.

In order to compare the quality of the impeller structure with other industrial applications, the results of the oxygen removal from water for another type of impellers were performed [35]. The view of all impellers is shown in Figure 6.

**Figure 7:**
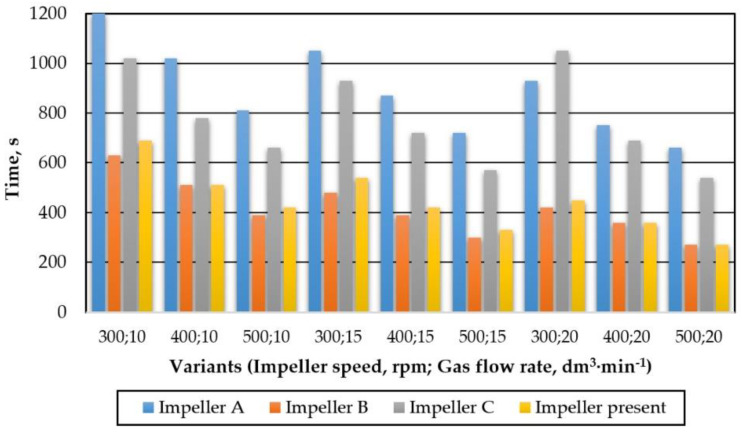
Summary of the refining times needed to remove 90% of dissolved oxygen for the tested variants for four different impellers.

Impellers A and B are typical pump type impellers, while C is a propeller-type impeller. In this case, tiny gas bubbles are generated by tiny holes in the impeller axis. The tested impeller in terms of operation is similar to the C-type rotor; however, it does not have as many sharp edges and bends as the C-type impeller. Figure 7 presents a summary of the refining times needed to remove 90% of dissolved oxygen for the tested variants for four different impellers.

The longest refining times were observed for impellers A and C, but for impeller C these times were slightly better. The best results were noted for impeller B. On average, this time was practically half as short as in the case of A. The impeller under test has a slightly higher refining time than impeller B. However, in some processing parameters—such as 400 rpm and 10 dm^3^·min^−1^; 400 rpm and 20 dm^3^·min^−^^1^; 500 rpm and 20 dm^3^·min^−1^—the times are almost the same. Comparing the tested impeller with the C impeller, which was also a propeller impeller, much better results (from 40 to even 50 %) of gas removal time were obtained, and at the same time the impeller had less sharp edges, which should significantly extend its service life.

**Figure 8:**
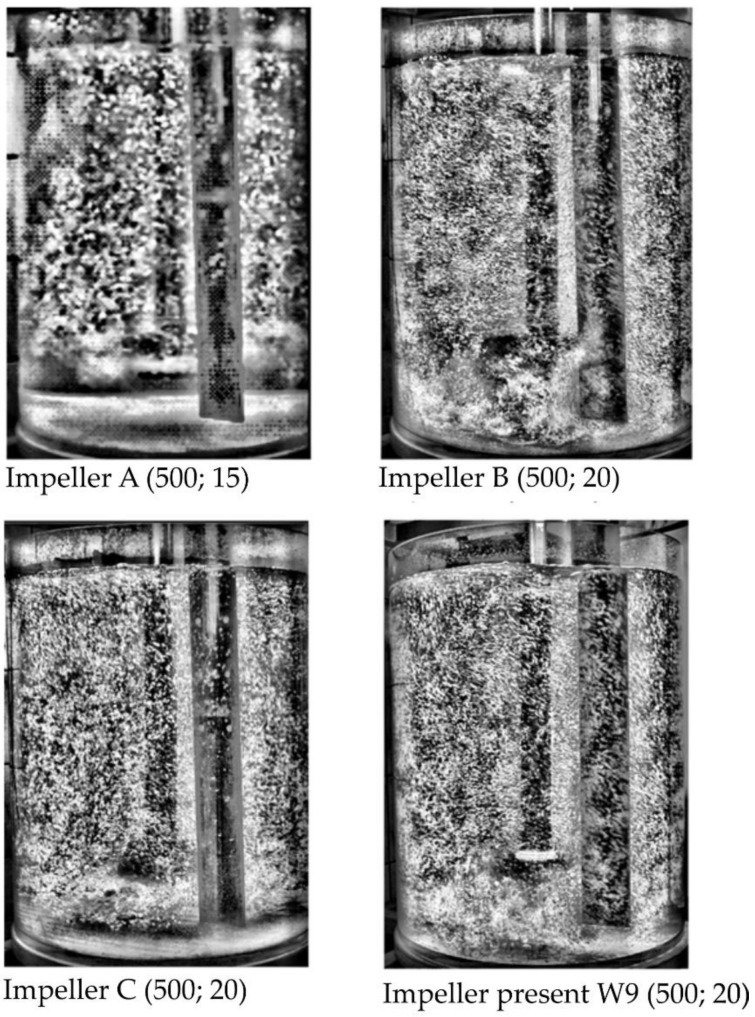
Results of the visualization research for four different types of impeller; in brackets, the impeller speed in rpm and the flow rate of gas in dm^3^·min^−1^.

Figure 8 presents a summary of the results of gas bubble dispersion visualization in the modeling liquid (water) for four different types of impellers (three of them described in detail in [35]) for the shortest refining times.

In the case of impeller A, there is no dispersion in the lower part of the reactor, and the gas bubbles generated are quite large (see Figure 8). When considering impeller B, excessive dispersion arises, and the chain bubbles are formed. In the case of impeller C, there is an intimate dispersion, because there are no gas bubbles and mixing in the lower part of the tank, while in the case of the tested impeller, excessive dispersion was found, but compared to the impeller B there was a greater chain production of gas bubbles.

## 5. Conclusions

Physical modeling is a good and helpful tool to better understand phenomena occurring during the refining process of aluminum and its alloys by purging them with argon. This is very important when considering a new impeller. In this case, it gives us the opportunity to choose the optimal processing parameters, such as the gas flow rate and the impeller speed, without expensive industrial research. The new designed impeller, in comparison with other impellers, is characterized by a good dispersion level of gas bubbles. Variants W5 (400 rpm, 15 dm^3^·min^−1^) and W7 (500 rpm, 10 dm^3^·min^−1^) seem to be the best processing parameters. Both are characterized by a uniform dispersion, and the refining time was 420 s. Variants W8 and W9 also yield promising results—the refining time was shorter (330 and 270 respectively), but excessive dispersions were observed, which means that the creation of a flow chain was observed, associated with the danger of reintroducing hydrogen into liquid aluminum. Considering that for the W8 and W9 variants the gas flow rate was 15 and 20 dm^3^·min^−1^, the W5 and W7 variants seem to be optimal for these types of impellers and are now recommended to be tested in the industry.

## Figures and Tables

**Table 1:** Comparison of the basic physical parameters and criterion numbers [33,35].

Parameter	Unit	Value	
Volume of the model	m^3^	0.23	
Characteristic dimension of the impeller	m	0.14	
Liquids		water	aluminum
Temperature	K	293	973
Kinematic viscosity	m^2^·s^−1^	1.005 · 10^−6^	1.000 · 10^−6^
Surface tension	N·m^−1^	0.072	0.868
Density	kg·m^−3^	1000	2700
Reynolds number	-	49,000	132,300
Weber number	-	238.19	53.35
Froude number	-	0.089	0.089

**Table 2:** Variants of the research conducted for different processing parameters.

No	Impeller Speed	Gas Flow Rate	No	Impeller Speed	Gas Flow Rate	No	Impeller Speed	Gas Flow Rate
	rpm	dm^3^·min^−1^		rpm	dm^3^·min^−1^		rpm	dm^3^·min^−1^
W1	300	10	W4	400	10	W7	500	10
W2		15	W5		15	W8		15
W3		20	W6		20	W9		20

**Table 3:** Summary of visualization results—different types of gas bubble dispersion.

Gas Flow Rate, dm^3^·min^−1^	Type of Dispersion/Impeller Speed, rpm		
	300	400	500
10	intimate	uniform	uniform
15	intimate	uniform	excessive
20	intimate	excessive	excessive

**Table 4:** Refining time to eliminate 90% of dissolved oxygen for studied variants.

Variant	Time, s	Variant	Time, s	Variants	Time, s
W1	690	W4	510	W7	420
W2	540	W5	420	W8	330
W3	450	W6	360	W9	270

## Data Availability

Data is contained within the article.
